# Multiomics Identification of Radioresistance‐Associated Biomarkers and Prognostic Model Construction in Rectal Cancer

**DOI:** 10.1155/humu/2759900

**Published:** 2026-05-22

**Authors:** Zongxueni Deng, Caiyan Lu, Zhenxin Wang

**Affiliations:** ^1^ Department of Oncology, The First Affiliated Hospital of Soochow University, Suzhou, China, sdfyy.cn

**Keywords:** bioinformatics, multiomics, nomogram, prognostic model, radioresistance, radiotherapy, rectal cancer

## Abstract

**Background:**

Radiotherapy remains a cornerstone in the local management of rectal cancer (RC); however, resistance to radiation significantly compromises therapeutic efficacy and adversely affects patient prognosis. Identification of biomarkers associated with radioresistance and the development of a prognostic model based on radiotherapy‐related genes in RC remain critical for enhancing treatment outcomes.

**Methods:**

Data related to RC were obtained from public repositories, including bulk RNA‐seq data from 993 patients across four Gene Expression Omnibus (GEO) and one The Cancer Genome Atlas (TCGA) cohort, as well as single‐cell RNA‐seq data from six samples. A prognostic model was developed using differential expression analysis, functional enrichment analysis, and least absolute shrinkage and selection operator regression analysis. Associations between risk score and prognosis were assessed through gene set variation analysis, gene set enrichment analysis, and construction of a nomogram to identify potential therapeutic targets for RC.

**Results:**

Prognosis‐related genes were determined through analysis of clinical data from patients with RC in the GEO and TCGA datasets, leading to the development of a risk score model. The risk score demonstrated significant associations with immune cell infiltration, chemotherapy drug sensitivity, and multiple signaling pathways. Protein expression levels of the key genes in patients with RC were verified using the Human Protein Atlas database. Furthermore, immunohistochemical evaluation in animal models provided additional validation.

**Conclusion:**

Molecular characteristics and mechanisms underlying radiotherapy response in RC were clarified through multiomics analysis. Five key genes were identified as potentially related to radiotherapy sensitivity in RC. These prognostic genes may serve as novel biomarkers and potential targets for diagnosis, prognostic evaluation, and clinical management of RC.

## 1. Introduction

With advancements in diagnostic techniques, therapeutic strategies, and overall living standards, rectal cancer (RC) has emerged as a highly prevalent malignancy, accounting for a significant proportion of colorectal cancer cases worldwide, with risk factors including obesity, lifestyle, and genetic predisposition [1, 2]. Surgical resection remains the primary treatment modality for RC and is often supplemented with chemotherapy, radiotherapy, targeted therapy, or immunotherapy depending on disease stage [1]. Radiotherapy plays a pivotal role in achieving local control of RC, particularly as a neoadjuvant treatment for RC, where it is aimed to reducing the risk of local recurrence and improve survival outcomes [1]. Mechanisms contributing to radioresistance in tumors include DNA damage repair, cell cycle arrest, apoptosis evasion, immune suppression, increased cancer stem cell populations, and alterations in both cancer cells and the tumor microenvironment [3].

Despite its role as a standard treatment for locally advanced RC, the effectiveness of neoadjuvant radiotherapy is frequently compromised by radioresistance of cancer cells, which adversely affects patient survival and contributes to treatment failure, disease progression, and poor prognosis [4]. Accordingly, identification of biomarkers associated with radioresistance is critical for evaluating therapeutic efficacy and monitoring tumor progression. In recent years, bioinformatics analysis has been increasingly utilized to analyze microarray and sequencing data for the identification of disease‐related genes and the exploration of their underlying mechanisms [5, 6]. The development of single‐cell sequencing and the widespread application of gene arrays have further facilitated the identification of key genes, providing valuable insights into guiding therapeutic strategies.

The aim of this study was to comprehensively examine RC through multidataset analysis, explore molecular mechanisms underlying radioresistance, and develop a prognostic model based on genes associated with radiotherapy‐related features in RC. This study tests the central hypothesis that this multiomics‐based prognostic model will provide superior predictive accuracy for clinical outcomes and serve as a reliable tool for guiding individualized therapeutic strategies in RC. This approach provides a novel framework for improving prognostic assessment and clinical outcomes in patients with RC undergoing radiotherapy.

## 2. Materials and Methods

### 2.1. Data Collection

Transcriptomic and clinical data were obtained from public repositories. Bulk RNA sequencing data were sourced from four Gene Expression Omnibus (GEO) datasets (accessed via https://www.ncbi.nlm.nih.gov/geo/), a public repository maintained by the National Center for Biotechnology Information (NCBI), and one The Cancer Genome Atlas (TCGA) dataset.

Single‐cell RNA sequencing (scRNA‐seq) data were derived from an additional GEO entry. The series matrix file for GSE35452 (Platform GPL570) included expression profiles from 46 patients (22 nonresponders and 24 responders to radiotherapy), GSE60331 (Platform GPL15207) contained data from 31 patients (16 nonresponders and 15 responders), GSE17536 (Platform GPL570) contained expression and survival data from 177 patients, and GSE39582 (Platform GPL570) comprised data from 562 patients with survival information. Additionally, processed RNA‐seq data and clinical information for the TCGA Rectal Adenocarcinoma (READ) project were obtained from the Genomic Data Commons Portal (https://portal.gdc.cancer.gov/), encompassing 177 patients. For single‐cell analysis, the dataset GSE278406, which provides complete single‐cell expression profiles from six tissue samples, was utilized.

### 2.2. Differential Expression Analysis

The Limma package in R was used for differential expression analysis to identify significantly differentially expressed genes (DEGs) between groups. The criteria for DEG identification were *p* < 0.05 and |logFC| > 0.585. Volcano plots and heatmaps were generated to visualize DEGs.

### 2.3. Functional Enrichment Analysis

Functional annotation of DEGs was performed using the Metascape database (http://www.metascape.org). Gene Ontology (GO) enrichment analysis was conducted to explore the molecular functions of the identified genes. Terms with an overlap of ≥ 3 genes and an adjusted ∗*p*∗value ≤ 0.01 were considered statistically significant. These thresholds were applied to ensure the biological relevance and statistical robustness of the enriched terms, as they help to filter out terms supported by only a few genes (which may be less reliable) and control for multiple testing, respectively.

### 2.4. Model Construction and Prognosis

A prognostic prediction model for RC was established using the least absolute shrinkage and selection operator (LASSO) regression analysis algorithm. Gene expression levels were incorporated, and an individualized risk score formula was calculated through regression analysis coefficient weighting. Patients were stratified into high‐risk and low‐risk groups based on the median score. Kaplan–Meier analysis, with log‐rank testing, was used to evaluate survival differences. Model performance was validated through stratified analysis and LASSO regression analysis, and predictive efficacy was assessed using receiver operating characteristic (ROC) curves.

### 2.5. Immune Cell Infiltration Analysis

The CIBERSORT algorithm, based on support vector regression analysis, was applied to evaluate immune cell types in the tumor microenvironment. The CIBERSORT algorithm, which deconvolves bulk expression profiles into 22 immune cell subsets—including T cells, B cells, plasma cells, and myeloid lineages—via 547 signature genes [7], was employed to quantify infiltrating populations and correlate them with gene expression. This analysis quantified the relative proportions of immune cell subsets and explored associations between gene expression and immune cell abundance.

### 2.6. Drug Sensitivity Analysis

Drug sensitivity prediction was performed using the Genomics of Drug Sensitivity in Cancer (GDSC) database (https://www.cancerrxgene.org). The R package “oncoPredict” estimated IC50 values for chemotherapy drugs using regression analysis methods, with 10‐fold cross‐validation applied to assess regression analysis accuracy. Batch effects were removed using the “combat” method, and duplicate gene expression values were averaged.

### 2.7. Gene Set Variation Analysis (GSVA)

GSVA, a nonparametric and sample‐level approach, was employed to quantify pathway enrichment. Hallmark gene sets (MSigDB 7.0) were first imported; enrichment scores per sample were then computed with the GSVA R package, converting gene‐level shifts into pathway scores to evaluate functional alterations [7].

### 2.8. Gene Set Enrichment Analysis (GSEA)

Based on the established risk scoring model, patients were divided into high‐risk and low‐risk subgroups. GSEA was applied to compare pathway differences between the groups using standard MSigDB gene sets as reference. Pathways with adjusted *p* values < 0.05 were considered statistically significant and ranked according to consistency scores.

### 2.9. Nomogram Model Construction

A nomogram was constructed as a visual prediction tool based on multivariate regression analysis. This tool used scaled line segments drawn proportionally on the same plane according to risk score expression levels and clinical features, thereby illustrating the relationships between variables in the prediction model. Contributions of each factor to the outcome variable were quantified by *β* coefficient magnitudes. Each level of the included factors was assigned a score, and predicted values were calculated through the summation of total scores.

### 2.10. Quality Control

Expression profiles were processed using the Seurat package. Cells were filtered based on total unique molecular identifier counts per cell, the number of expressed genes, and the mitochondrial gene expression proportion per cell. The mitochondrial gene expression proportion was defined as the ratio of mitochondrial‐encoded gene expression to the total transcriptome expression. Since mitochondrial gene expression proportion is negatively correlated with RNA abundance, high values indicate initiation of cell mortality programs. Quality control was performed using the median absolute deviation, with data exceeding three times the typically considered outliers. Additionally, potential doublets were identified and removed using the DoubletFinder algorithm.

### 2.11. Data Normalization and Cell Annotation

Gene expression data were normalized using the LogNormalize method, setting the total expression of each cell to 10,000 followed by log‐transformation. Cell cycle activity was evaluated using the CellCycleScoring function, whereas highly variable genes were identified with FindVariableFeatures. ScaleData was applied to correct for mitochondrial and ribosomal gene expression as well as cell cycle effects. RunPCA was used for linear dimensionality reduction, and RunUMAP was applied for nonlinear dimensionality reduction. Batch effects were corrected using Harmony. Cell type annotation was performed using evidence from published literature, the CellMarker database, and SingleR software to identify disease‐related cell populations.

### 2.12. Ligand–Receptor Interaction Analysis

To map intercellular communication, we used CellChat [7] on the scRNA‐seq data; interaction weights and counts were computed as previously described. This method integrates network analysis and pattern recognition algorithms to characterize signal transduction and functional regulatory relationships among cell subpopulations. Standardized single‐cell expression profiles were combined with cell subtype information identified through single‐cell clustering analysis to construct intercellular interaction networks. Two indicators, interaction weights and interaction counts, were calculated to assess the strength of communication among different cell subpopulations.

### 2.13. Protein Expression Validation via the Human Protein Atlas

To validate the protein expression levels of uromodulin‐like 1 (UMODL1), phospholamban (PLN), mesothelin (MSLN), and homeobox C6 (HOXC6) in RC and corresponding normal tissues, we interrogated the Human Protein Atlas (HPA) (http://www.proteinatlas.org). For each gene, we examined all available immunohistochemistry (IHC) data for “colorectal cancer” and “normal tissue—colon/rectum.” Protein expression levels were primarily determined according to the HPA′s own scoring system, which integrates staining intensity and the proportion of positive cells. Representative images provided by the HPA were used as a visual reference. Based on the above assessment, expression was categorized into four levels: “high,” “medium,” “low,” or “not detected.”

### 2.14. Cell Culture

The human male colorectal adenocarcinoma cell line HCT116 (*Homo sapiens*, male, colon adenocarcinoma [COAD], RRID:CVCL_0291) was purchased from Procell Life Science & Technology Co., Ltd. (Wuhan, China) in 2025. Cells were cultured in McCoy′s 5A medium supplemented with 10% fetal bovine serum and maintained at 37°C in a humidified atmosphere containing 5% CO_2_. Mycoplasma contamination was tested before the experiments. The cell line was verified to be free of mycoplasma and authenticated by short tandem repeat (STR) profiling.

### 2.15. Ionizing Radiation (IR)

The radiation source used in this study is located at the Irradiation Center of the School of Radiation Medicine, Soochow University, which emits x‐rays. A single‐fraction irradiation protocol was applied with the following parameters: a source‐to‐surface distance of 50 cm, a dose rate of 1 Gy/min, and a total absorbed dose of 4 Gy.

### 2.16. Animal Experiments

This study utilized ten 5‐week‐old female BALB/c nude mice (athymic) for the xenograft tumor experiment. The mice were purchased from Cyagen (Suzhou) Bioscience Co., Ltd. and housed in a specific pathogen‐free (SPF) environment at the Soochow University Animal Experiment Center (License No. XCYK [Su] 2002‐0008). For the cell‐derived xenograft (CDX) model, HCT116 cells in the logarithmic growth phase (5 × 10^6^ cells per mouse) were injected subcutaneously into the nude mice. One week after tumor cell inoculation, tumor volume was measured using a vernier caliper to confirm tumor engraftment. Mice that failed to form tumors or exhibited abnormal tumor volumes (either too large or too small) were excluded. The remaining tumor‐bearing mice were then randomly divided into two groups (*n* = 5 per group) using a random number table method: a control group (CTRL) and an irradiation group (IR). When the tumor volume reached approximately 100 mm^3^, a single‐fraction irradiation protocol was administered: Local irradiation to the axillary region was performed with lead shielding, using a source‐to‐skin distance of 50 cm, a dose rate of 1 Gy/min, and a total absorbed dose of 4 Gy.

### 2.17. IHC Staining

IHC staining was performed on formalin‐fixed, paraffin‐embedded (FFPE) tumor tissue sections (4 *μ*m thick) from nude mouse xenografts. Following deparaffinization in xylene and rehydration through a graded ethanol series, antigen retrieval was conducted by heating the slides in sodium citrate buffer (pH 6.0). Endogenous peroxidase activity was blocked with 3% hydrogen peroxide, and nonspecific binding sites were blocked with normal goat serum. The sections were then incubated overnight at 4°C with primary antibodies against MSLN (Zenbio, Catalog No. R382355; dilution 1:200), PLN (Zenbio, Catalog No. R25354; dilution 1:150), HOXC6 (ABMART, Catalog No. PA400S; dilution 1:100), and UMODL1 (ABMART, Catalog No. PH15617S; dilution 1:100). After washing, the sections were incubated with corresponding horseradish peroxidase (HRP)–conjugated secondary antibodies (Zenbio) at room temperature for 1 h. The immunoreaction was visualized using a 3,3 ^′^‐diaminobenzidine (DAB) chromogen substrate, followed by counterstaining with hematoxylin. Finally, the sections were dehydrated, cleared, and mounted. Stained images were observed and captured under an Olympus IX81 microscope. Protein expression levels were assessed based on staining intensity and the percentage of positive cells.

### 2.18. Statistical Analysis

Survival curves were generated using the Kaplan–Meier method, and between‐group differences were assessed with the log‐rank test. Multivariate analysis was conducted using the Cox proportional hazards regression analysis model. All statistical analyses were performed using R software (Version 4.3.0). Statistical significance was defined as *p* < 0.05.

Where analytical procedures overlapped with those reported by Mu et al. [7], we adhered to their published protocols and cite the source accordingly.

## 3. Results

### 3.1. PCA Correction and Differential Analysis

**Figure 1:**
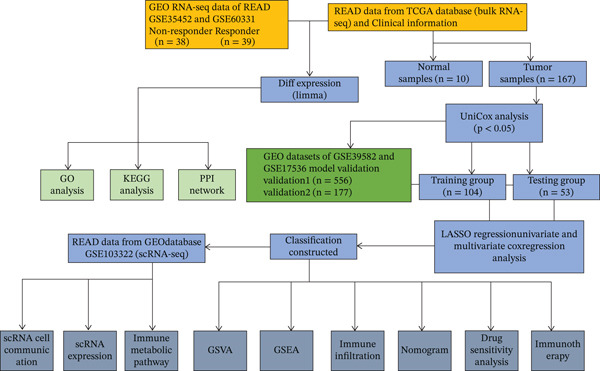
Research patients selection and GEO and TCGA data analysis process of this study.

The flowchart shows the research patients selection, GEO, and TCGA data analysis process of this study (Figure 1). Datasets GSE35452 and GSE60331, comprising 77 RC samples (38 nonresponder and 39 responder cases), were obtained from the NCBI GEO public database. Batch effects in the microarray data were corrected using the surrogate variable analysis (SVA) algorithm. Principal component analysis (PCA) visualization demonstrated that after SVA processing, distribution differences between batches were reduced (Figure 2a, b). DEGs were identified using the Limma package, applying criteria of *p* < 0.05 and |logFC| > 0.585.

Figure 2(a, b) PCA after batch effect removal. (c) Volcano plot of DEGs (red: upregulated genes, *n* = 31; blue: downregulated genes, *n* = 69). (d) Heatmap of DEGs between radiotherapy‐resistant (green) and responsive (orange) groups (blue: low expression; red: high expression). (e) Visualization of protein–protein interaction network for DEGs. (f) Enrichment analysis results of DEGs.

**Figure:**
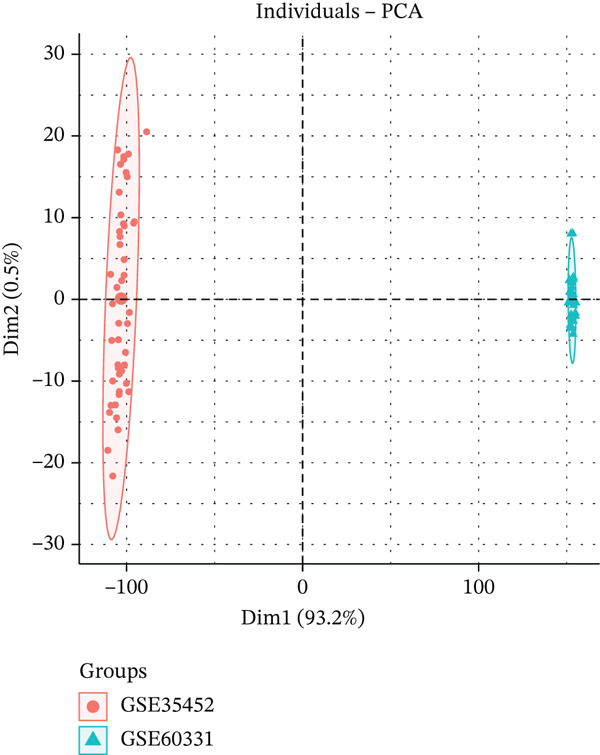
(a)

**Figure:**
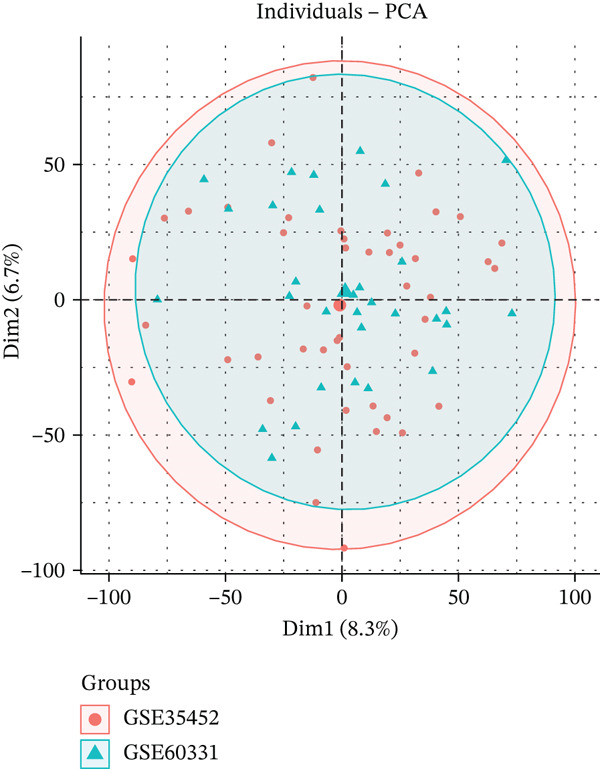
(b)

**Figure:**
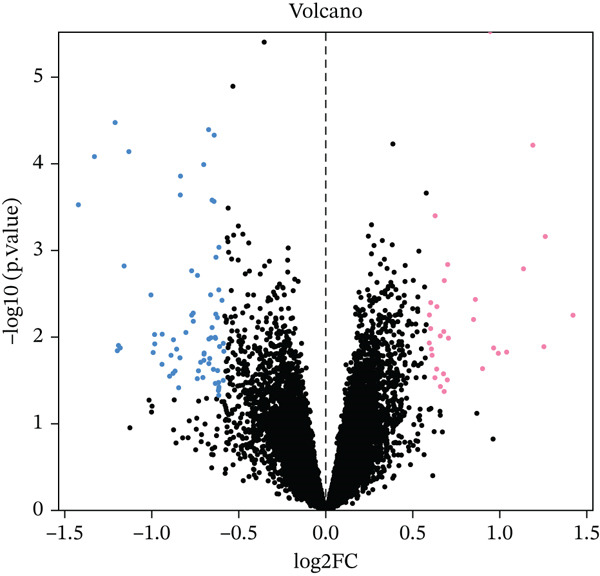
(c)

**Figure:**
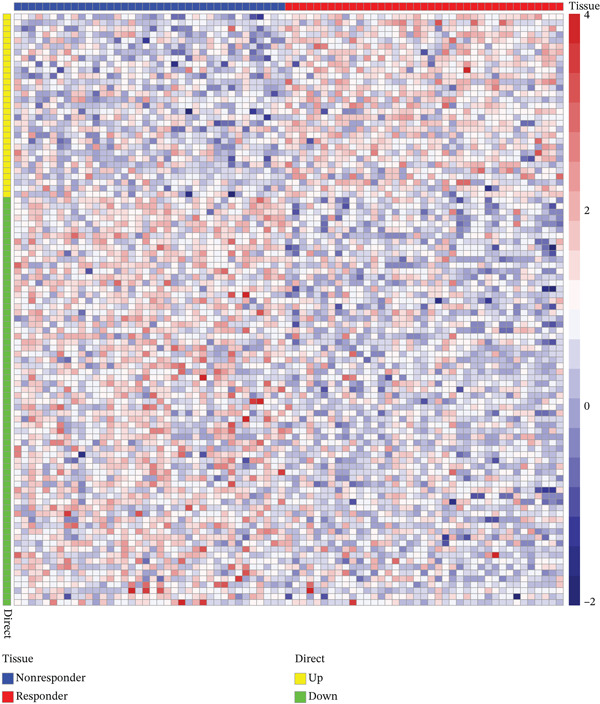
(d)

**Figure:**
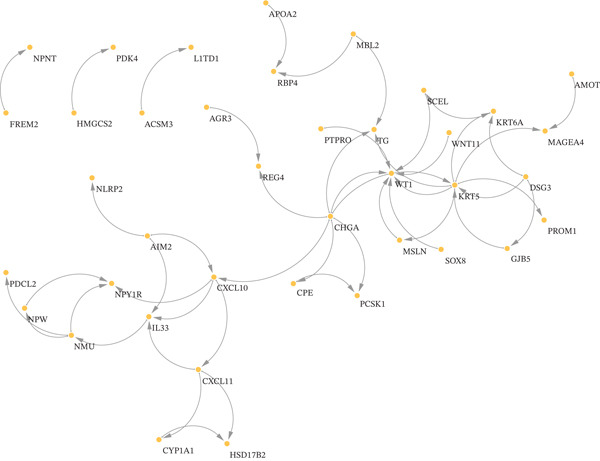
(e)

**Figure:**
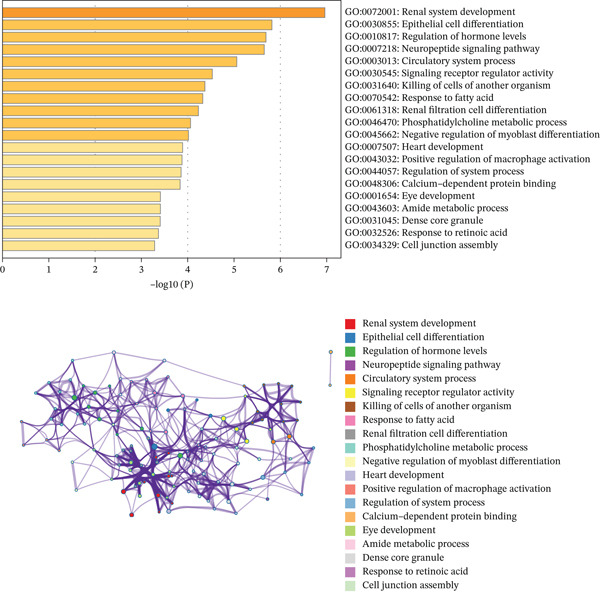
(f)

A total of 100 DEGs were identified, including 31 upregulated and 69 downregulated genes (Figure 2c, d). Protein–protein interaction networks of the DEGs were constructed using the STRING database (https://cn.string-db.org/) and http://cn.string-db.org/ visualized with Cytoscape software (Figure 2e). Pathway enrichment analysis through the Metascape database indicated that these genes were predominantly enriched in epithelial cell differentiation, circulatory system processes, phosphatidylcholine metabolic processes, and related pathways (Figure 2f).

### 3.2. Construction of Prognostic Model

Clinical data of patients with RC from the TCGA database were analyzed, and prognostic genes were screened from the identified DEGs using Cox univariate regression analysis. Six prognosis‐related genes (*p* < 0.05) were identified (Figure 3a). Based on the LASSO regression analysis algorithm, five key genes with the highest predictive value were selected. The TCGA dataset containing survival data was randomly divided into training and test sets at a 2:1 ratio. Following LASSO regression analysis (Figures 3b, c, and d), optimal risk score values were calculated using the following formula: Risk score = CCDC85A × 0.0848430925884313 + MSLN × 0.319483666791475 + UMODL1 × 0.331564570543034 + PLN × 0.396571430580948 + HOXC6 × 0.543131661348868. Patients were classified into high‐risk and low‐risk groups according to the median risk score. Kaplan–Meier survival analysis demonstrated shorter overall survival (OS) in the high‐risk group compared with the low‐risk group in both training and test sets (Figure 3e, f). ROC curve analysis confirmed good predictive performance in both sets (Figure 3g, h).

Figure 3(a) Univariate Cox regression analysis identified six genes significantly associated with rectal cancer prognosis. (b) LASSO coefficient profiles of the candidate prognostic genes. (c) Cross‐validation was performed to determine the optimal lambda value in the LASSO regression model. (d) Regression coefficients of the selected genes and their corresponding log2 (HR) values.(e) Kaplanâ€“Meier survival curve for the training dataset. (f) Kaplanâ€“Meier survival curve for the testing dataset. (g) Time‐dependent ROC curves for 1‐, 3‐, and 5‐year survival in the training dataset. (h) Time‐dependent ROC curves for 1‐, 3‐, and 5‐year survival in the testing dataset.

**Figure:**
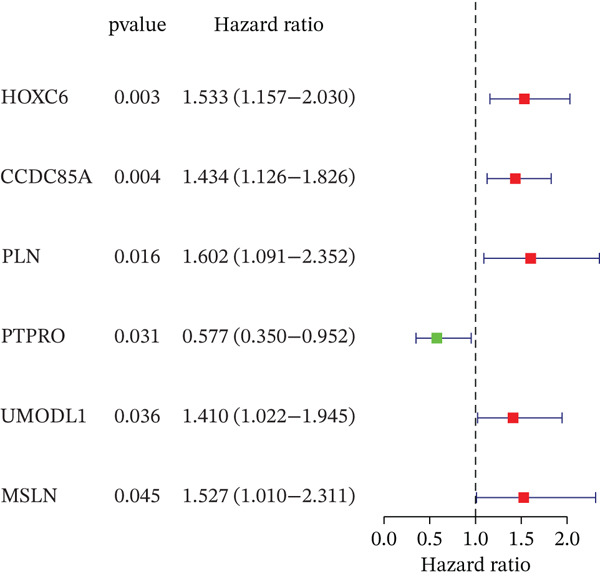
(a)

**Figure:**
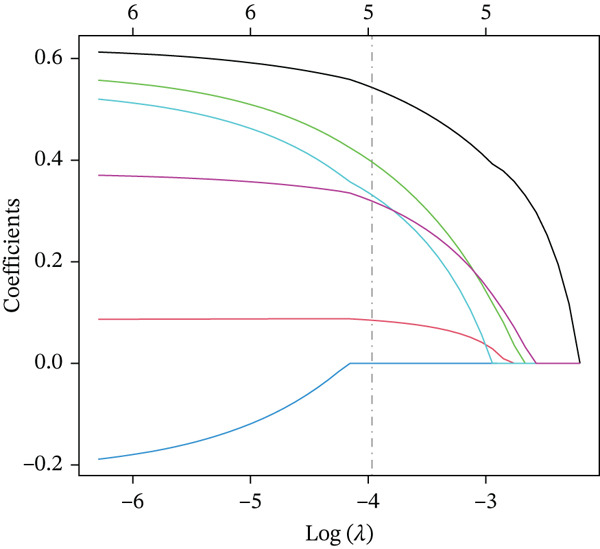
(b)

**Figure:**
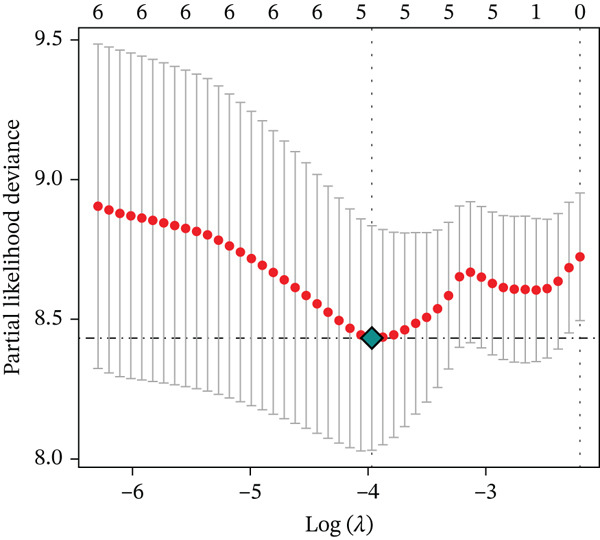
(c)

**Figure:**
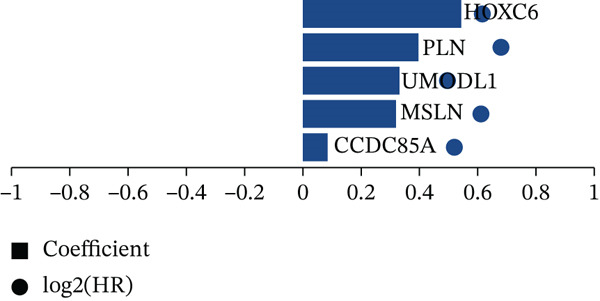
(d)

**Figure:**
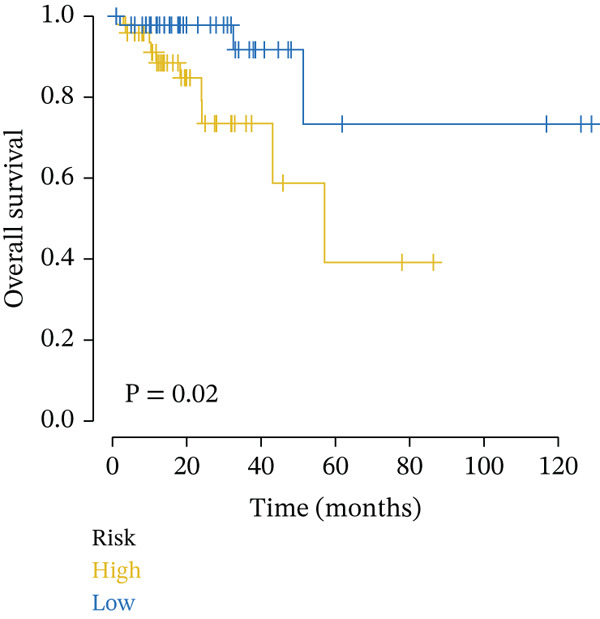
(e)

**Figure:**
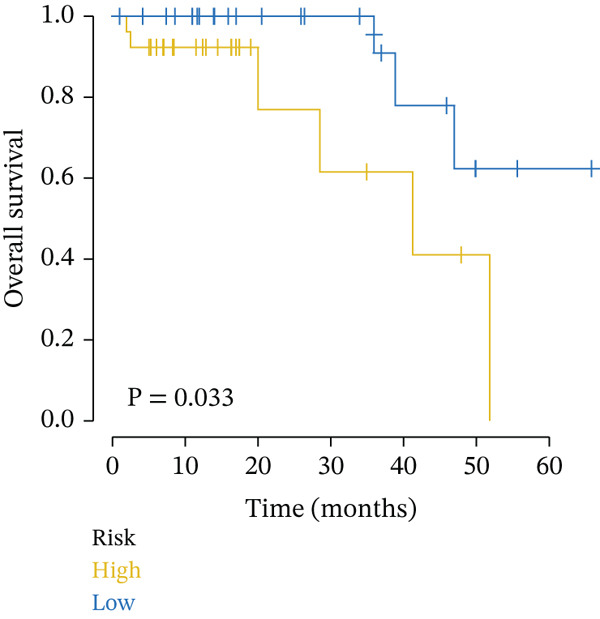
(f)

**Figure:**
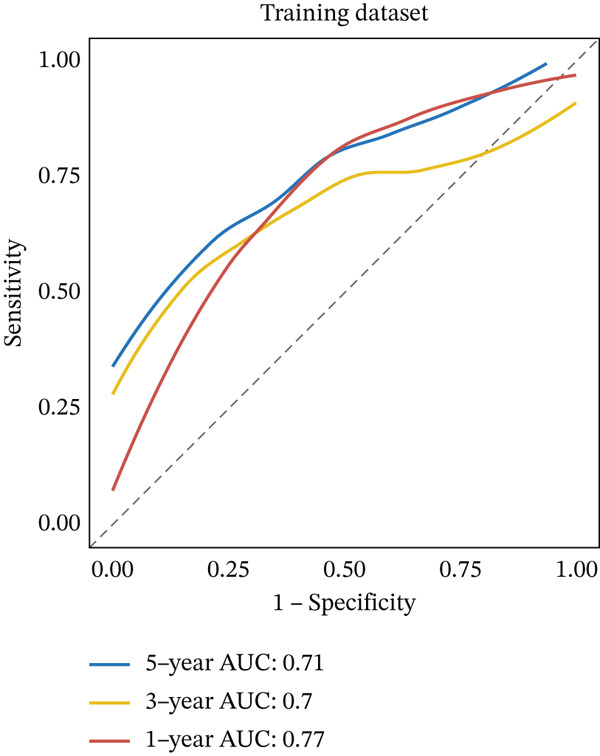
(g)

**Figure:**
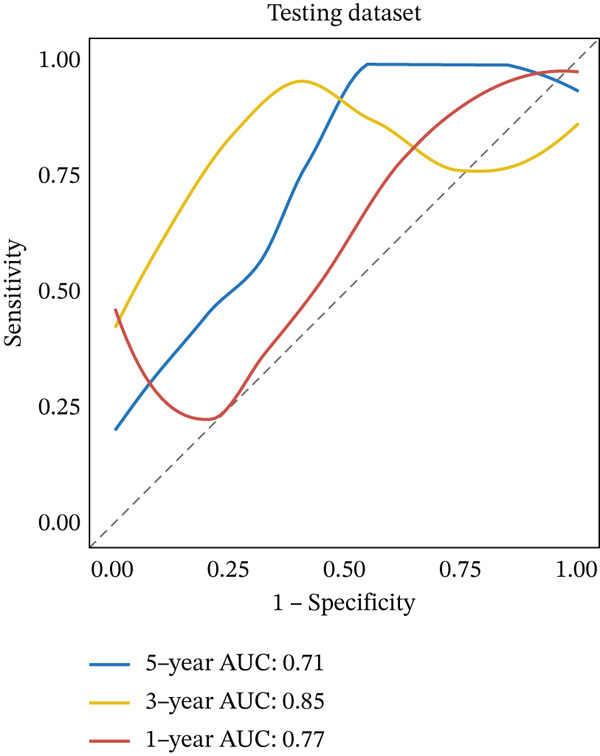
(h)

### 3.3. Robustness of External Validation

External validation was performed using RC patient data with survival information from GSE17536 to GSE39582 in the GEO database. Patients were stratified into high‐risk and low‐risk groups according to the risk model. Kaplan–Meier analysis indicated that OS was lower in the high‐risk group than in the low‐risk group across external datasets (Figure 4a, b, b). ROC curve analysis further confirmed that the model maintained strong predictive efficacy for prognosis in external validation cohorts (Figure 4c, d).

Figure 4(a) Kaplan–Meier survival curves showing the difference in overall survival between high‐ and low‐risk groups (*p* = 0.004). (b) Kaplan–Meier survival curves showing the difference in overall survival between high‐ and low‐risk groups in another cohort (*p* = 0.006). (c) ROC curves for Validation Dataset 1, with AUC values for 1‐, 3‐, and 5‐year survival predictions of 0.65, 0.62, and 0.60, respectively. (d) ROC curves for Validation Dataset 2, with AUC values for 1‐, 3‐, and 5‐year survival predictions of 0.65, 0.66, and 0.64, respectively.

**Figure:**
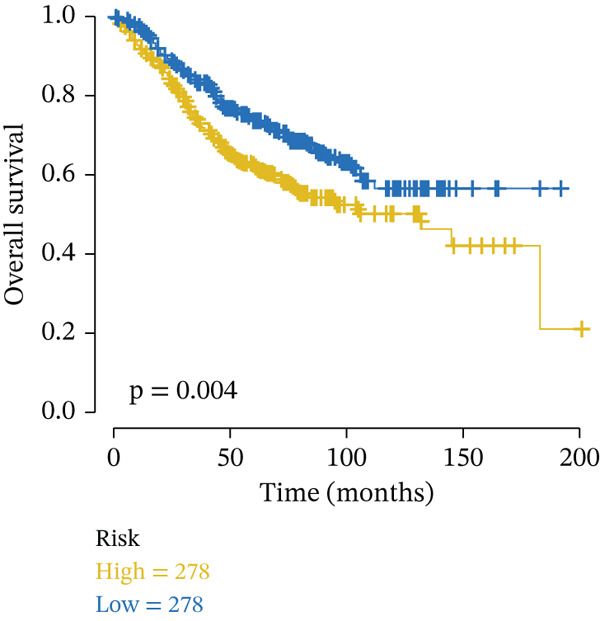
(a)

**Figure:**
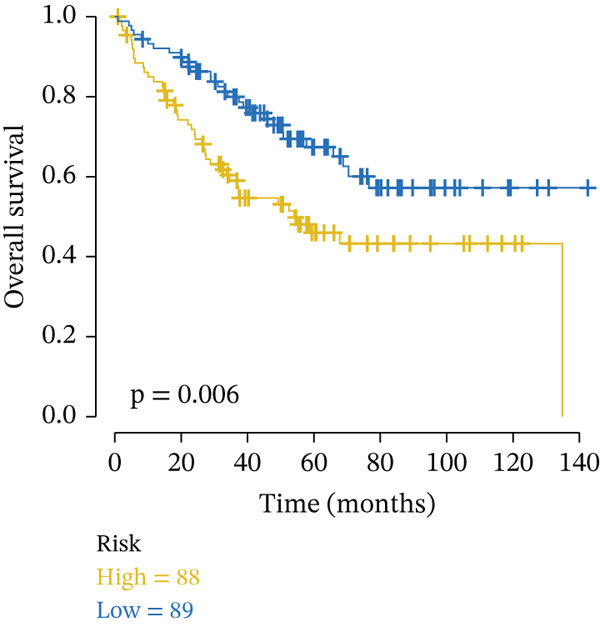
(b)

**Figure:**
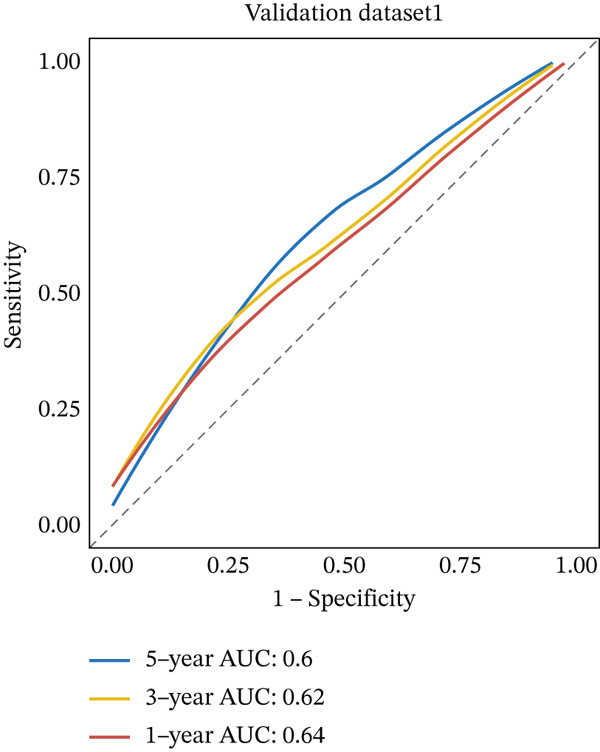
(c)

**Figure:**
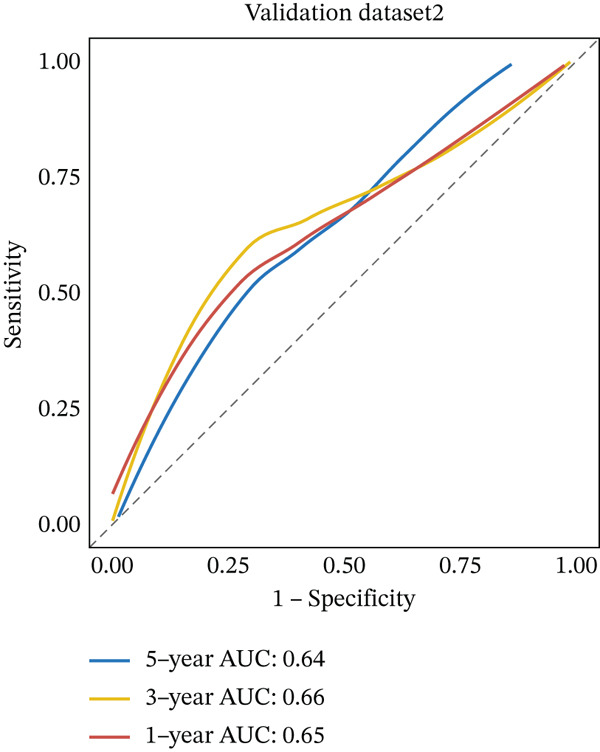
(d)

### 3.4. Immune Infiltration

The tumor microenvironment, composed of tumor cells, fibroblasts, immune cells, extracellular matrix, and various cytokines and chemokines, exerts critical influence on tumor initiation, progression, diagnostic classification, and prognosis. Associations between risk score and tumor immune infiltration were analyzed. The proportions of immune cell subsets in high‐risk and low‐risk groups are presented in Figure 5a, b. Significant differences in immune cell abundance were observed between groups, particularly for M0 macrophages, M2 macrophages, monocytes, and neutrophils (Figure 5c). Correlation analysis demonstrated that the risk score was significantly positively associated with M2 macrophages and M0 macrophages and significantly negatively associated with activated CD4 memory T cells and plasma cells (Figure 5d).

Figure 5(a) Percentage of immune cells between high‐ and low‐risk groups, green: high‐risk group and purple: low‐risk group. (b) Interaction analysis between different immune cells in high‐ and low‐risk group READ patients ( ^∗^ represents *p* < 0.05,  ^∗∗^ represents *p* < 0.01,  ^∗∗∗^ represents *p* < 0.001. (c) Comparison of immune cells between low‐risk and high‐risk groups, blue: low risk group and purple: high‐risk group. (d) Distribution of specific immune cell types—M0 macrophages, M1 macrophages, M2 macrophages and plasma cells—between the high‐ and low‐risk groups.

**Figure:**
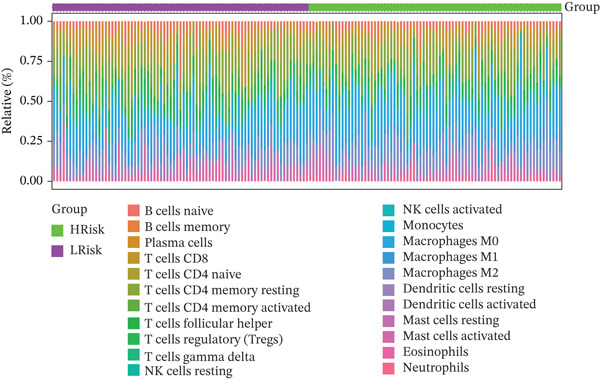
(a)

**Figure:**
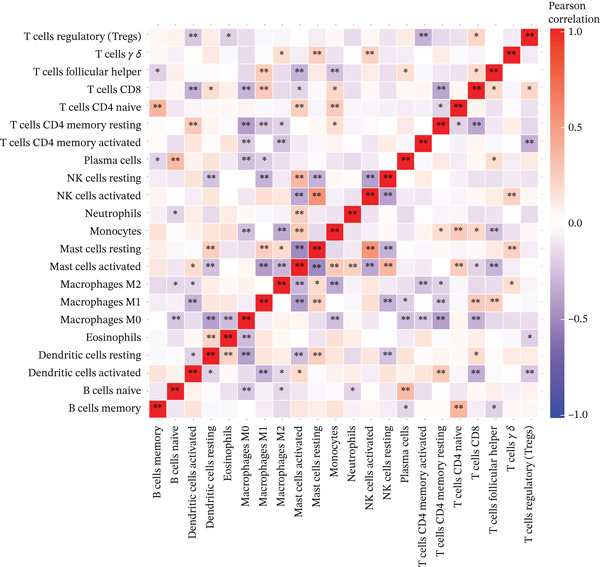
(b)

**Figure:**
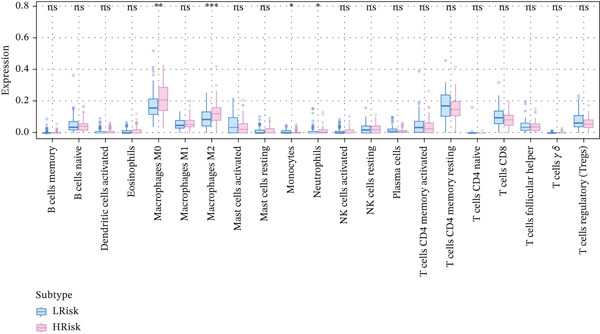
(c)

**Figure:**
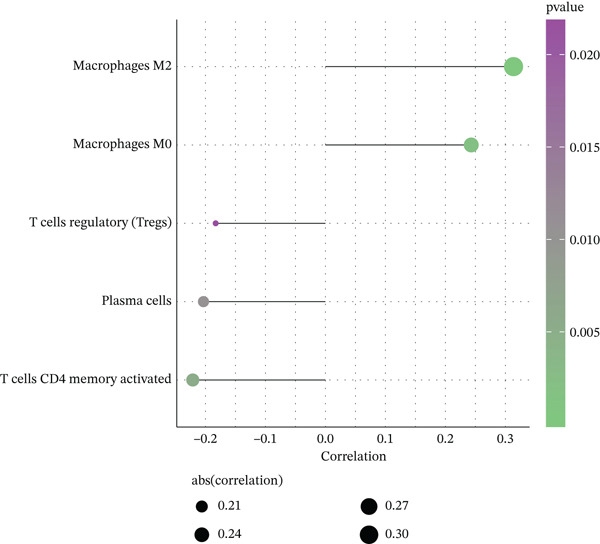
(d)

### 3.5. Drug Sensitivity Analysis and Signaling Pathways Involving Risk Score

Although surgery combined with adjuvant chemoradiotherapy is standard treatment for early RC, therapeutic efficacy varies among patients. To evaluate this heterogeneity, drug sensitivity was predicted using the GDSC database and the R package “oncoPredict.” Significant associations were observed between risk score and sensitivity to 5‐Fluorouracil_1073, Sorafenib_1085, Irinotecan_1088, Oxaliplatin_1089, Erlotinib_1168, and Temozolomide_1375 (Figure 6a). GSVA revealed enrichment of pathways such as TGF_BETA_SIGNALING, HEDGEHOG_SIGNALING, and NOTCH_SIGNALING in high‐ versus low‐risk groups (Figure 6b). GSEA further identified pathways including Wnt signaling, PI3K‐Akt signaling, and phagosome processes (Figure 6c). Molecular interaction networks among these pathways are presented in Figure 6d.

Figure 6(a) Correlation between high/low‐risk groups and drug sensitivity. (b, c) GSVA and GSEA reveal signaling pathways enriched by differentially expressed genes between high/low‐risk groups. (d) Molecular interaction network between signaling pathways.

**Figure:**
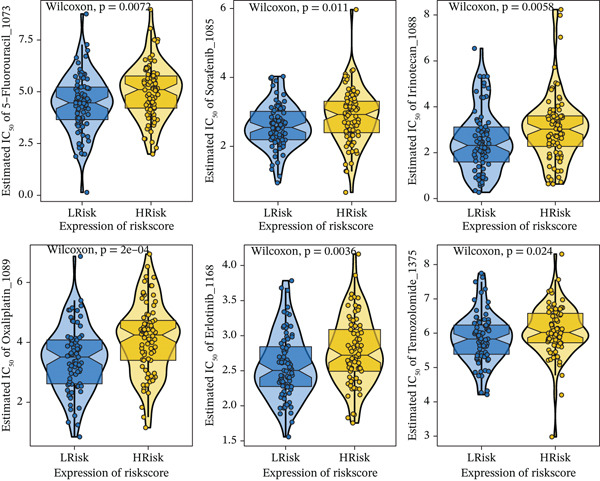
(a)

**Figure:**
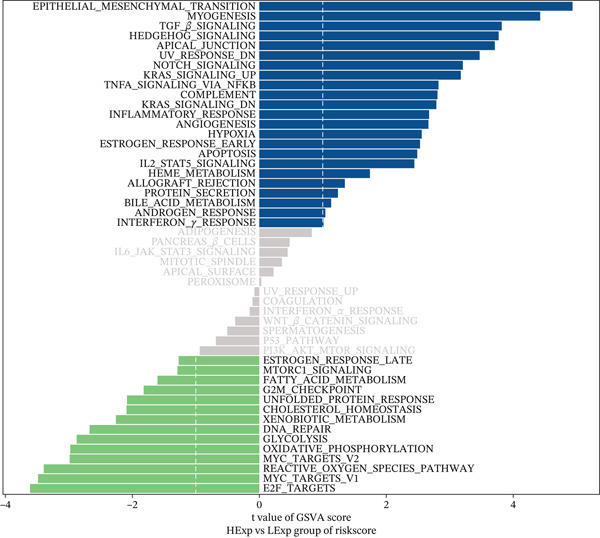
(b)

**Figure:**
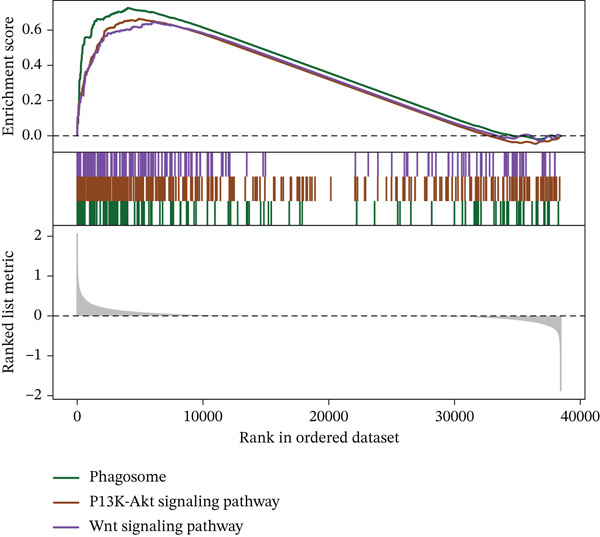
(c)

**Figure:**
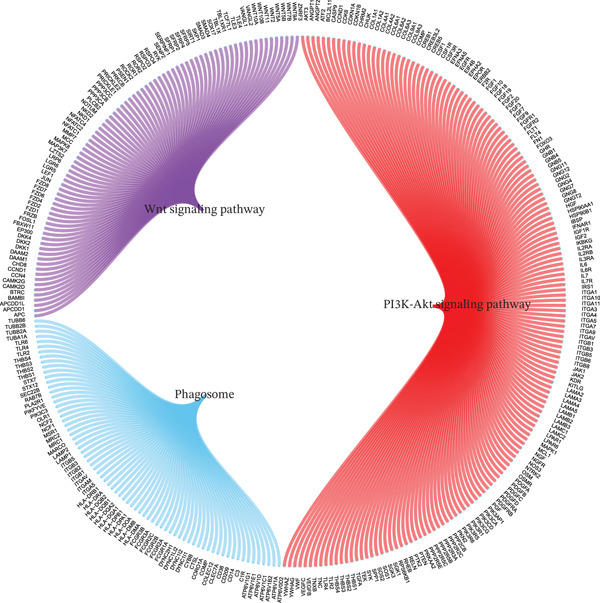
(d)

### 3.6. Nomogram Model Prediction

Patients were divided into high‐risk and low‐risk groups according to the median risk score. A predictive model was constructed using multivariate regression analysis, and a nomogram was developed to visually estimate 1‐, 3‐, and 5‐year overall survival probabilities (Figure 7a). Calibration analysis demonstrated good agreement between predicted and observed survival outcomes (Figure 7b). Model performance was further evaluated using ROC and decision curve analysis (DCA) (Figure 7c,d). The sensitivity of the risk score to antitumor immunotherapy was also assessed, indicating that the immunotherapy response rate was significantly lower in the high‐risk group than in the low‐risk group (Figure 7e).”

Figure 7(a) Nomogram predicts individual 1‐, 3‐, and 5‐year survival probabilities. (b) Calibration curve validates model accuracy. (c) ROC curve demonstrates model performance in 1‐, 3‐, and 5‐year survival predictions, with AUC values of 0.8243, 0.8392, and 0.8024, respectively. (d) DCA curve compares predictive efficacy of risk score with other clinical variables. (e) Response rates to immunotherapy in high‐ versus low‐risk groups.

**Figure:**
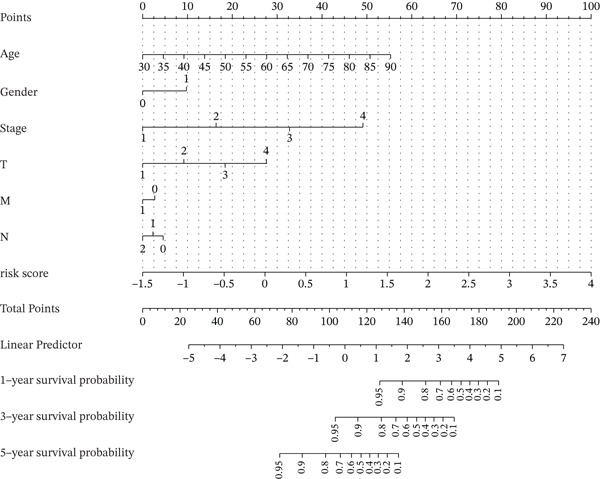
(a)

**Figure:**
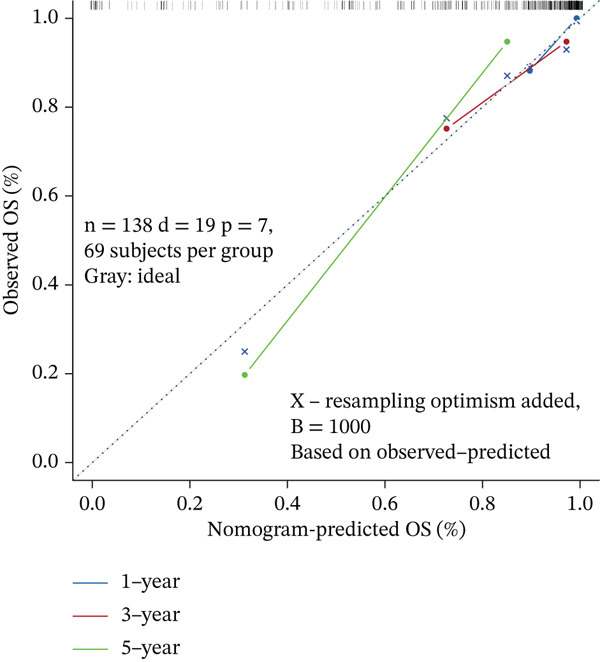
(b)

**Figure:**
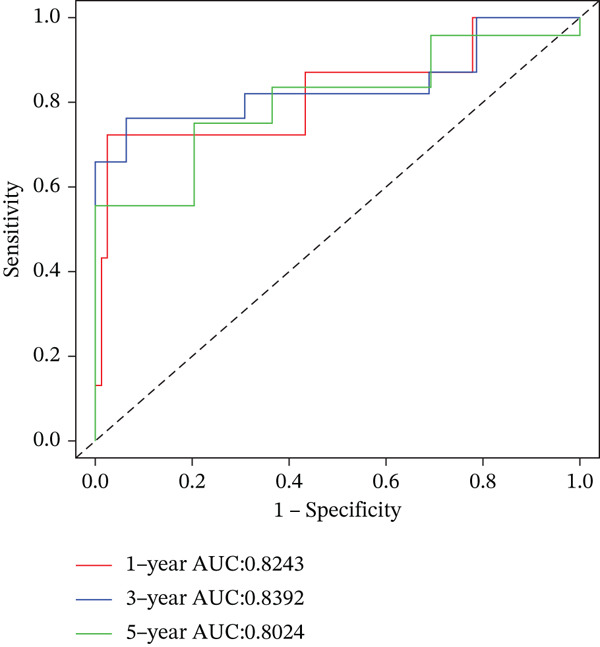
(c)

**Figure:**
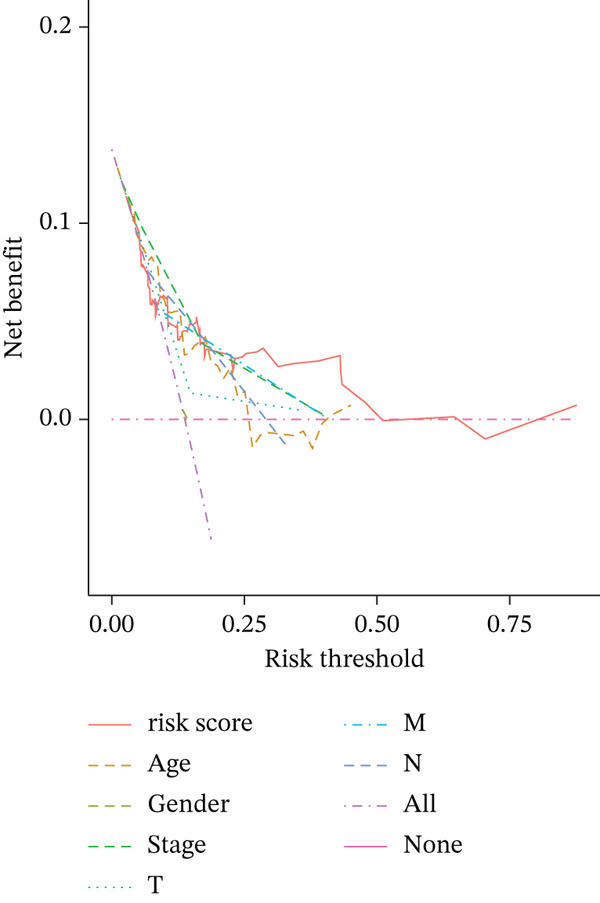
(d)

**Figure:**
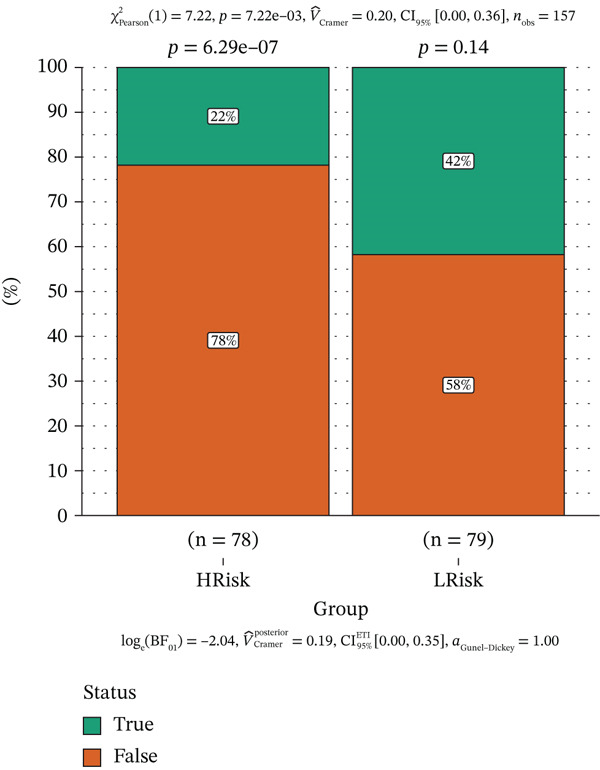
(e)

### 3.7. Single‐Cell Data Quality Control

To ensure data quality across multiple samples, cells with fewer than 200 detected genes and other outliers were removed, resulting in 45,350 retained cells (Figure S1). Postfiltering violin plots and scatter plots are presented in Figure S2a,b. A total of 2000 highly variable genes were identified, and the Top 10 genes with the highest standard deviation were displayed (Figure S2c). Data were processed through normalization, scaling, PCA, and Harmony‐based correction (Figures S2d, 2e, f).

### 3.8. Cell Annotation and Cell Communication Analysis

Cell annotation using established cell markers identified 10 subtypes (Figure 8a and Table S1). These were grouped into seven categories: CD8 T cells, monocytes, neutrophils, B cells, CD4 T cells, cell cycle cells, and dendritic cells (Figure 8b). Classic marker expression for these seven cell types is presented in bubble plots (Figure 8c), with corresponding cell proportion distributions displayed in pie charts (Figure 8d). Ligand–receptor relationships were analyzed using the CellChat package, revealing complex intercellular interaction networks among these subtypes (Figure 8e). Monocytes exhibited more pronounced intercellular interaction characteristics compared with other immune cell subsets (Figure 8f, g).

Figure 8(a) UMAP clustering identifies 10 cell subtypes (color‐coded). (b) Annotated into seven major cell types (e.g., T cells and B cells). (c) Bubble plot shows marker expression (percentage by size and average level by color). (d) Pie chart displays cell type proportions in sample. (e) Left shows cell subtype interactions, right shows interaction strength. (f) Total interactions per cell type. (g) Ligand–receptor interaction strength (color: coexpression; size: *p*‐value).

**Figure:**
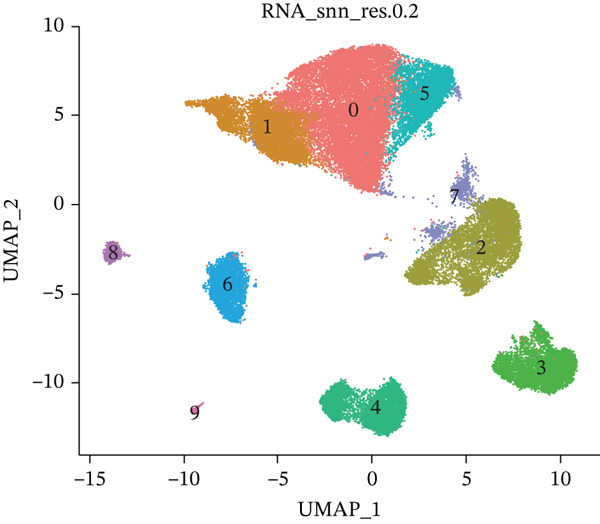
(a)

**Figure:**
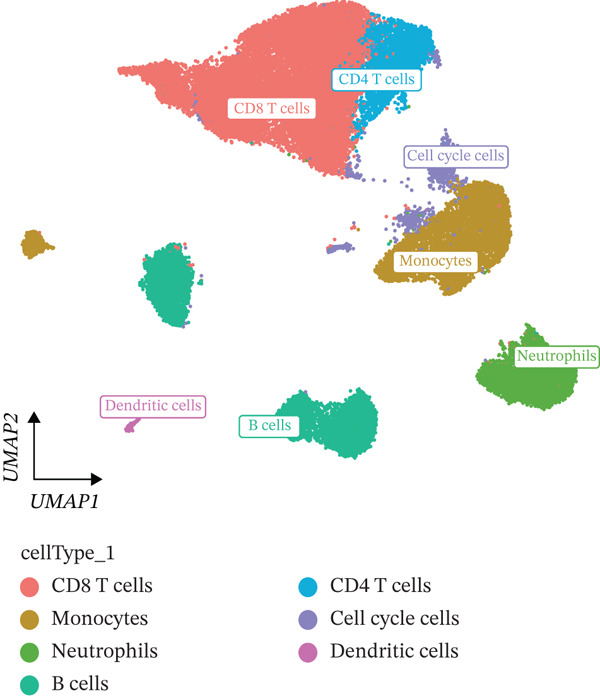
(b)

**Figure:**
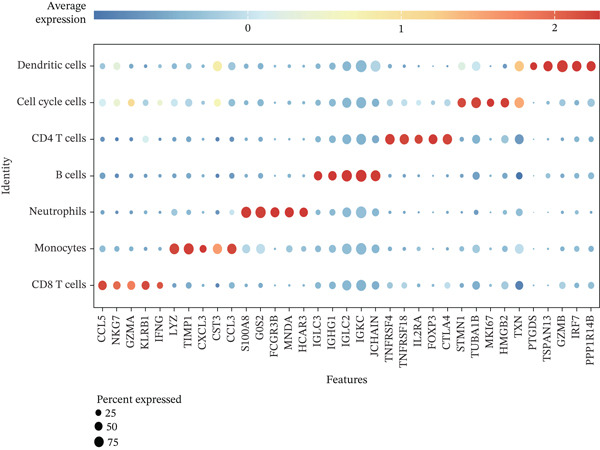
(c)

**Figure:**
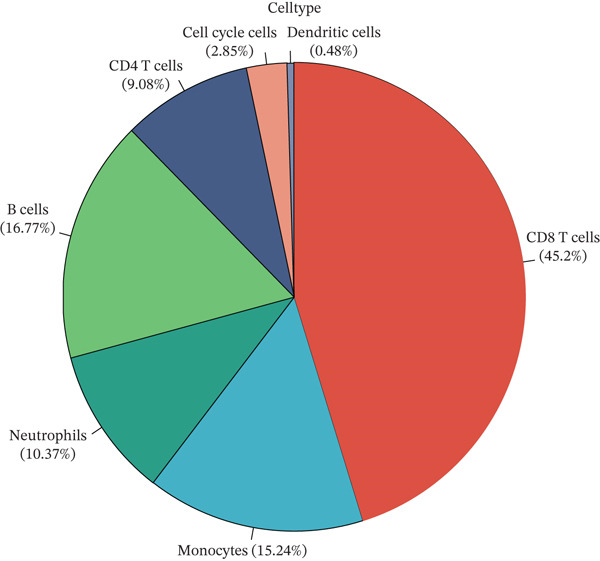
(d)

**Figure:**
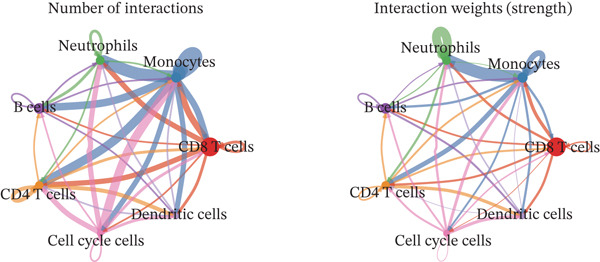
(e)

**Figure:**
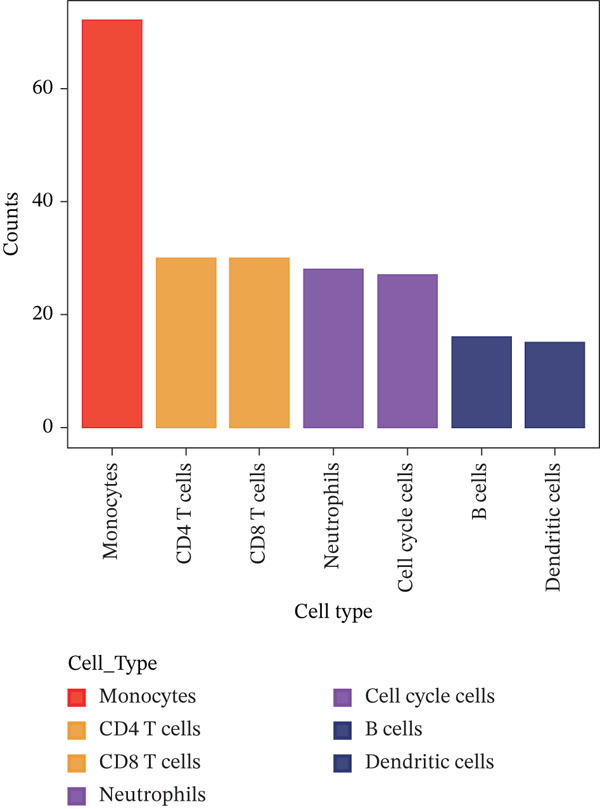
(f)

**Figure:**
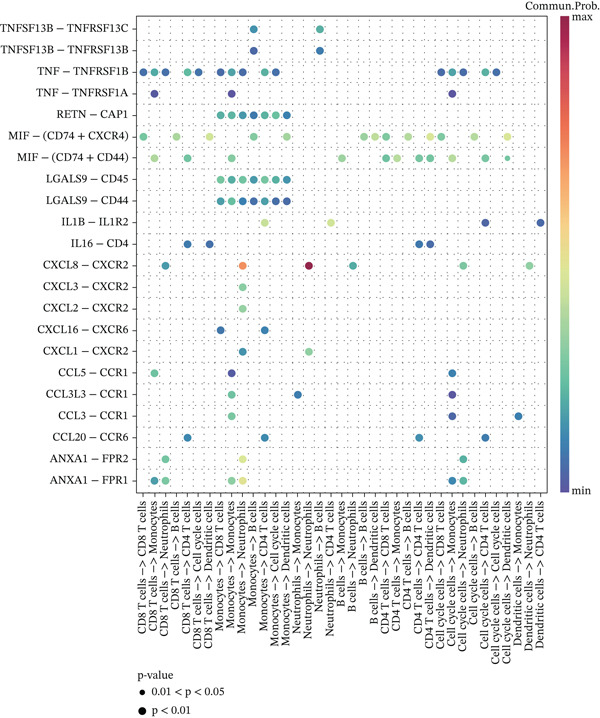
(g)

### 3.9. Expression Profiles of Model Genes in Single Cells

Expression analysis of key genes across the seven identified cell types demonstrated that *CCDC85A* was significantly expressed in B cells, *MSLN* in cell cycle cells, *UMODL1* in cell cycle cells, *PLN* in CD4 T cells, and *HOXC6* in B cells (Figure 9a, b). The AUCell function was used to quantify immune and metabolic pathway activities at the single‐cell level. Bubble plots illustrated pathway activity differences associated with key genes. *UMODL1* exhibited high activity in oxidative phosphorylation and MYC_TARGETS_V1 pathways, *PLN* in oxidative phosphorylation and MYC_TARGETS_V1 pathways, *MSLN* in TNFA_SIGNALING_VIA_NFKB and UNFOLDED_PROTEIN_RESPONSE pathways, *HOXC6* in G2M_CHECKPOINT and MYC_TARGETS_V1 pathways, and *CCDC85A* in UNFOLDED_PROTEIN_RESPONSE and MYC_TARGETS_V1 pathways (Figure 9c).

Figure 9(a) Expression levels of key genes in seven cell types. (b) UMAP plot showing expression distribution of key genes across cells. (c) Activity of key genes in different biological pathways.

**Figure:**
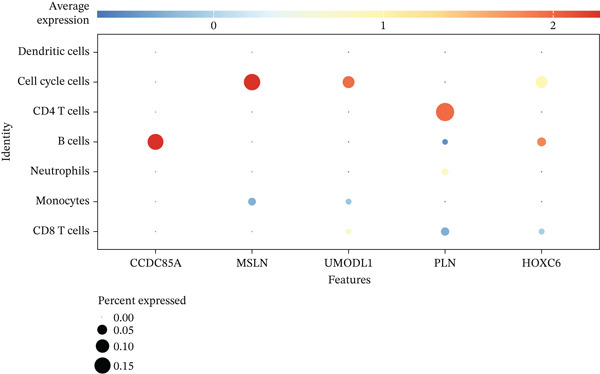
(a)

**Figure:**
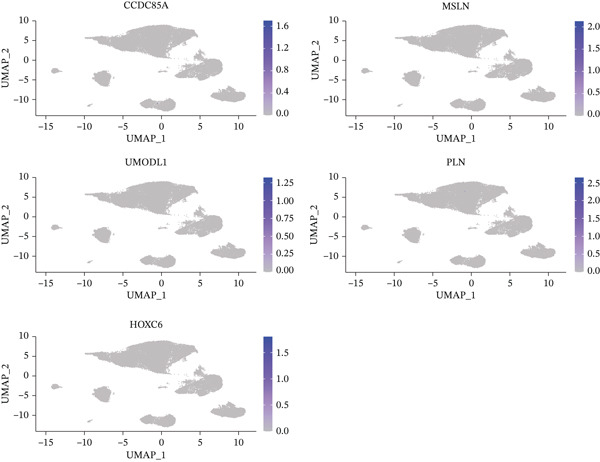
(b)

**Figure:**
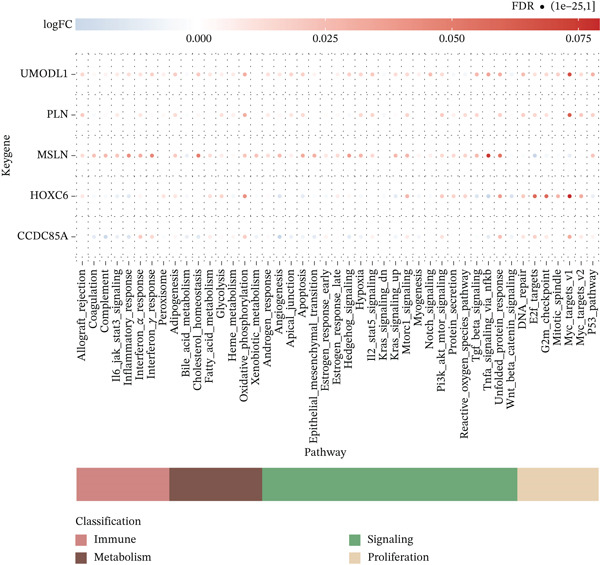
(c)

### 3.10. Validation of Model Gene Expression Levels in Clinical Samples and Animal Models

IHC results from the HPA are presented in Figure 10a. *UMODL1* was absent in normal rectal tissue and demonstrated absent or low expression in RC. *PLN* showed minimal expression in both rectal tissue and RC. *MSLN* exhibited moderate expression in normal rectal tissue and variable expression in RC, ranging from low to high. *CCDC85A* demonstrated high expression in normal rectal tissue and moderate to high expression in RC. *HOXC6* expression data were unavailable in the HPA database. Variability in gene expression was influenced by biological heterogeneity among patients with RC and differences in radiosensitivity.

Figure 10(a) Protein expression levels of key genes in rectal tissue and colorectal cancer from the HPA database. (b) Immunohistochemical images of key genes from paraffin‐embedded samples in nude mice, scale bar = 50 *μm* (applies to all images). (CTRL: control group; IR: 4Gy irradiated group).

**Figure:**
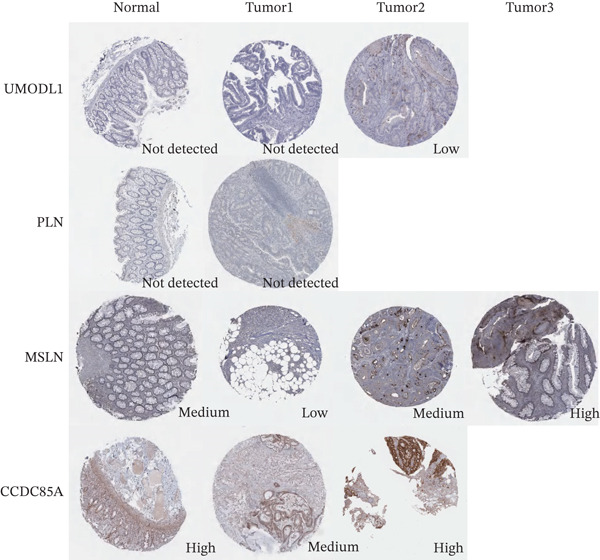
(a)

**Figure:**
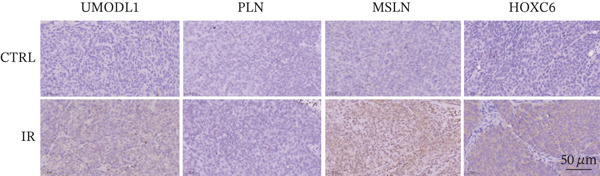
(b)

To further assess the role of prognostic genes in radiotherapy sensitivity, a human colorectal cancer cell (HCT116) nude mouse xenograft (CDX) model was established. Nude mice were subjected to 4 Gy local irradiation, and IHC was performed to assess expression of the five prognostic genes. Results indicated upregulation of UMODL1, MSLN, and HOXC6 in the irradiation group, with MSLN and HOXC6 demonstrating more pronounced changes, whereas PLN demonstrated no significant change (Figure 10b). These findings support the relevance of key genes in RC radioresistance; however, further investigation is required to elucidate the underlying mechanisms.

## 4. Discussion

RC is a prevalent malignancy of the digestive tract worldwide, with incidence continuing to increase due to population aging and lifestyle changes [2]. Although treatment strategies combining surgery, radiotherapy, chemotherapy, targeted therapy, and immunotherapy have improved patient prognosis, recurrence and metastasis remain major contributors to treatment failure [8, 9]. Consequently, the development of reliable prognostic assessment models is essential for RC clinical management. Accurate prediction of disease risk enables clinicians to design individualized treatment plans, provide precise prognostic consultation, and ultimately improve both survival outcomes and quality of life [10]. The present study applied multiomics analysis to establish a novel prognostic risk assessment model for RC. The model effectively distinguished between high‐risk and low‐risk patients and demonstrated stability and accuracy across multiple datasets, offering a valuable tool for precise diagnosis and treatment planning in RC.

The risk score model exhibited robust prognostic predictive efficacy across independent datasets, indicating high applicability and reliability as a prognostic assessment tool. Furthermore, pharmacogenomic analysis using the GDSC database demonstrated significant correlations between risk scores and chemotherapy drug sensitivity. These findings provide potential guidance for optimizing chemotherapy regimen selection and underscore the importance of integrating individualized risk scores into clinical decision‐making. Further investigations should clarify the molecular mechanisms linking risk scores and chemotherapy responses to enhance therapeutic strategies for RC.

From the TCGA dataset, five key genes significantly associated with prognosis were identified. *CCDC85A*, a member of the coiled‐coil domain‐containing (CCDC) protein family, plays diverse roles in tumor biology [11, 12]. Immunohistochemical analyses in gastric and pancreatic cancers revealed that *CCDC85A* is variably expressed by cancer cells and cancer‐associated fibroblasts (CAFs). Regulation by miR‐224‐3p has been reported to enhance cancer cell tolerance to endoplasmic reticulum stress [13]. However, the function of *CCDC85A* in RC remains insufficiently defined.

MSLN, a cell surface glycoprotein, is highly expressed in several malignancies, including mesothelioma, epithelial ovarian cancer, pancreatic ductal adenocarcinoma, and gastric adenocarcinoma, while being minimally expressed in normal organ parenchyma [14]. MSLN has been implicated in enhancing tumor cell adhesion, proliferation, migration, and invasion, as well as in promoting epithelial–mesenchymal transition and acquisition of cancer stem cell traits, contributing to gemcitabine resistance in triple‐negative breast cancer [15]. Additionally, MSLN expression has been associated with cholangiocarcinoma, endometrial cancer, and lung cancer [15, 16]. However, evidence regarding the role of MSLN in RC radioresistance remains lacking.

*UMODL1*, located on chromosome 21q22.3, has been associated with conditions such as hyposmia, severe myopia, premature ovarian insufficiency, and ovarian degeneration [17, 18]. In oncology, *UMODL1* expression is generally low, and abnormal methylation has been identified as a hallmark of cancer. Specifically, aberrant methylation of *UMODL1/OIT3* has been linked to poor prognosis in COAD. *UMODL1* has also been identified as a predictive target gene within the LINC00114/miR‐216a‐5p axis colorectal cancer prognostic model and has demonstrated close associations with COAD survival [19]. Nevertheless, its role in mediating RC radiotherapy sensitivity remains undetermined.

The transcription factor *HOXC6* has been reported to be highly expressed in several solid tumors, including RC, esophageal squamous cell carcinoma, pancreatic cancer, and oral cancer [20–23]. In RC, *HOXC6* has been implicated in promoting metastasis via regulation of the DKK1/Wnt/*β*‐catenin signaling axis [24]. Additionally, *HOXC6* expression has been negatively correlated with *MLH1*, a core DNA mismatch repair (MMR) protein. Functional loss of *MLH1* inactivates the MMR pathway, resulting in diminished DNA repair capacity, which has been demonstrated to influence cancer cell radiosensitivity [25]. These findings indicate that *HOXC6* may contribute to DNA damage repair after RC radiotherapy, though specific mechanisms remain unclear.

*PLN*, an endogenous inhibitor of sarcoplasmic reticulum Ca^2+^‐ATPase, is predominantly distributed in cardiac tissue. Under physiological conditions, *PLN* regulates calcium pump activity via cyclic AMP‐dependent mechanisms, thereby mediating positive inotropic responses of the myocardium [26]. In pathological states, elevated *PLN* expression suppresses ATP1A2 activity, contributing to heart failure [27]. In RC, elevated *PLN* expression has been associated with tumor progression within the consensus molecular subtype 4 (CMS4) classification [28]. To date, no studies have examined the role of PLN in RC radioresistance.

It is noteworthy that the in vivo validation in this study was conducted using a COAD‐derived cell line (HCT116). Although this model provided an experimentally tractable system for initial proof‐of‐concept testing, we recognize that employing patient‐derived RC‐specific models in future work would enhance the direct translational relevance of our findings to RC.

Although our study provides a multiomics‐based prognostic framework, several limitations must be acknowledged. Firstly, the sample size, particularly in the external validation cohort, remains limited, which may affect the statistical power and generalizability of the model. Secondly, the inherent heterogeneity of data sourced from different public repositories (TCGA and GEO), despite rigorous bioinformatic normalization, may introduce residual batch effects or confounding factors. Most importantly, the current research is primarily based on computational analysis and retrospective clinical correlations. The lack of direct in vitro and in vivo functional validation experiments for the identified key genes restricts our ability to establish the causal mechanisms underlying their association with radiotherapy response.

To address these shortcomings, future studies could be pursued in the following directions: validation of this prognostic model in larger, prospectively designed, multicenter clinical cohorts is needed to genuinely assess its clinical translational potential. Subsequently, functional experiments, such as gene knockdown/overexpression combined with radiosensitivity assays (e.g., clonogenic survival assays), are crucial for clearly defining the functional roles of CCDC85A, MSLN, UMODL1, HOXC6, and PLN in mediating radioresistance.

## 5. Conclusion

Through integrated multiomics analysis, we identified and validated a five‐gene prognostic signature associated with radiotherapy response in RC. This signature effectively stratified patients into distinct risk groups and demonstrated robust predictive performance across independent cohorts. Furthermore, we constructed a clinically interpretable nomogram that integrates molecular risk with key clinical variables, offering a practical tool for individualized prognosis assessment. Our study not only elucidates potential molecular mechanisms underlying radioresistance but also provides candidate biomarkers and targets that may guide future therapeutic strategies and improve clinical outcomes in RC.

## Author Contributions

**Zongxueni Deng:** conceptualization, software, writing – original draft. **Caiyan Lu:** validation, formal analysis, data curation, writing – review and editing. **Zhenxin Wang:** resources, writing – review and editing, funding acquisition.

## Funding

This work was supported by the National Natural Science Foundation of China (82473566) and Jiangsu Provincial Science and Technology Programs (BE2022728).

## Ethics Statement

All experiments were evaluated and approved by the Ethics Committee of Soochow University (No. 202304A0198). Principles of Laboratory Animal Care (NIH Publication Vol. 25, No. 28 revised 1996; http://grants.nih.gov/grants/guide/notice-files/not96-208.html) were followed, as well as specific national laws (e.g., the current version of the German Law on the Protection of Animals) where applicable [29].

## Conflicts of Interest

The authors declare no conflicts of interests.

## Supporting Information

Additional supporting information can be found online in the Supporting Information section.

## Supporting information


**Supporting Information 1** Figures S1–S2: DoubletFinder doublet removal efficiency across samples (Figure S1) and scRNA‐seq quality‐control embeddings with top variable genes (Figure S2).

**Supporting Information 2** Table S1: Marker genes used for cell type annotation in scRNA‐seq analysis. Both files will be available online alongside the final article.

## Data Availability

The data that support the findings of this study are available in GEO at https://www.ncbi.nlm.nih.gov.com/geo/info/datasets.html. These data were derived from the following resources available in the public domain: Gene Expression Omnibus (https://www.ncbi.nlm.nih.gov/geo/info/datasets.html) and The Cancer Genome Atlas (https://portal.gdc.cancer.gov/).
