# Supplementary material for: Multiomics Identification of Radioresistance‐Associated Biomarkers and Prognostic Model Construction in Rectal Cancer
Source: Hum Mutat. 2026 May 22;2026:2759900. doi: 10.1155/humu/2759900 (PMC13197572; doi:10.1155/humu/2759900)
Supplement: Supplementary file 1 — Supporting Information 1 Figures S1–S2: DoubletFinder doublet removal efficiency across samples (Figure S1) and scRNA‐seq quality‐control embeddings with top variable genes (Figure S2). [file HUMU-2026-2759900-s002.doc]

**Supplement**

**Supplementary Figure 1.** Visualization of doublet detection in six scRNA-seq samples using DoubletFinder. Red dots denote doublets, blue dots singlets. This approach enhances data accuracy by identifying doublets for removal.


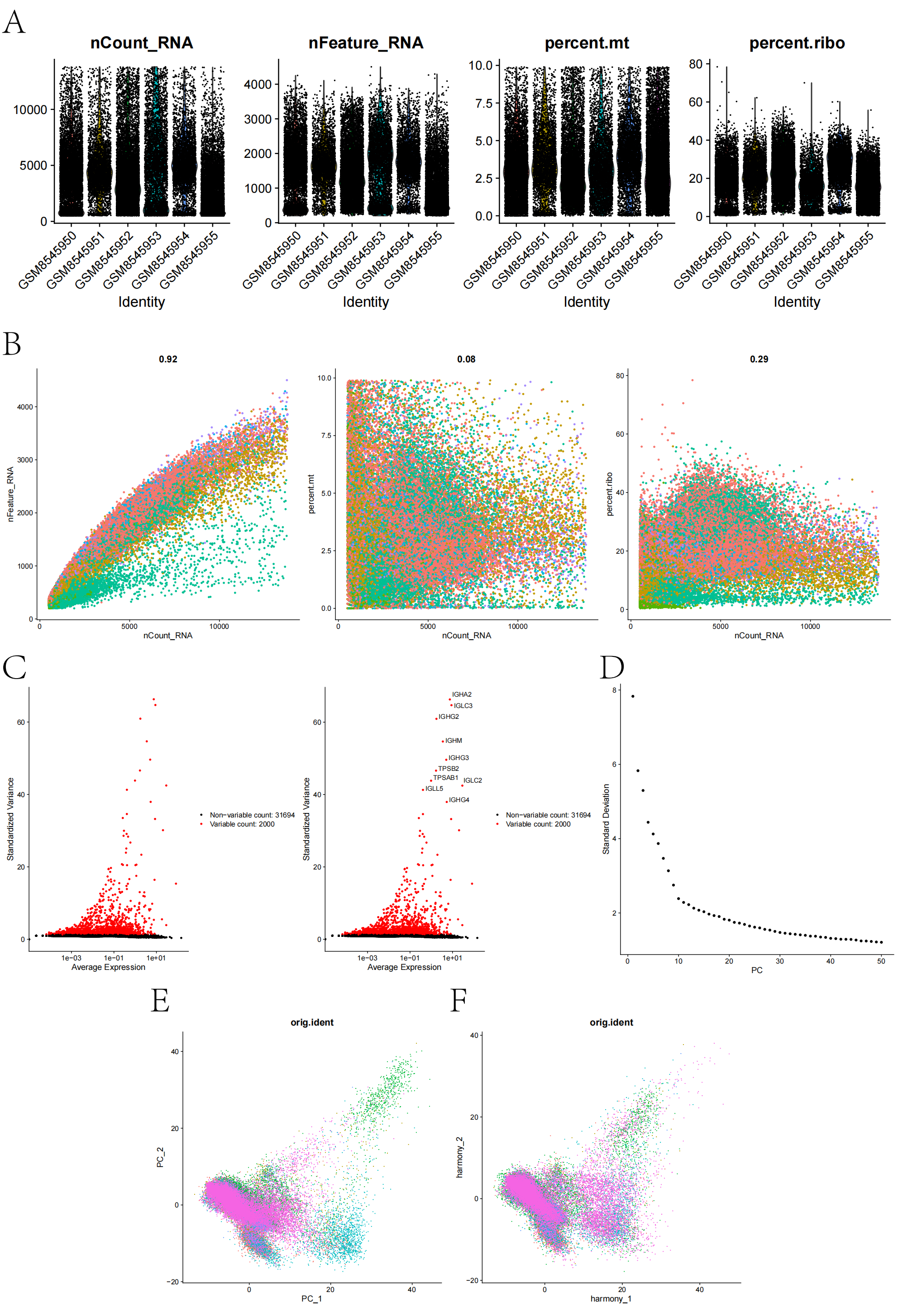


**Supplementary Figure 2.** (A–B) Violin and scatter plots after quality control, demonstrating 45,350 cells retained. (C) Top 10 highly variable genes with the greatest standard deviation (e.g., *IGHA2*, *IGLC3*, *IGHG2*). (D) Distribution of average expression for the top 10 highly variable genes after standardization and normalization. (E–F) PCA and Harmony results demonstrating uniform sample distribution along PC1 axis after batch effect removal.
